# Assessing Long-Term Exposure in the California Teachers Study

**Published:** 2011-06

**Authors:** Bart Ostro, Peggy Reynolds, Debbie Goldberg, Andrew Hertz, Richard T. Burnett, Hwashin Shin, Edward Hughes, Cynthia Garcia, Katherine D. Henderson, Leslie Bernstein, Michael Lipsett

**Affiliations:** California Office of Environmental Health Hazard Assessment, Oakland, California, E-mail: Bostro@Creal.cat; Cancer Prevention Institute of California, Berkeley, California; Health Canada, Ottawa, Ontario, Canada; Edward Hughes Consulting, Ottawa, Ontario, Canada; California Air Resources Board Sacramento, California; City of Hope, Duarte, California; California Department of Public Health, Richmond, California

In an article published in *Environmental Health Perspectives* (Ostro et al. 2010), we analyzed the relationships of long-term exposure to fine particulate matter (≤ 2.5 μm in aerodynamic diameter; PM_2.5_) and its components with mortality in a cohort of > 100,000 active and retired female professionals participating in the California Teachers Study (CTS) cohort. We used a Cox proportional hazards model in which pollution exposure was measured as a continuous variable over the study period. Monthly average pollutant concentrations were obtained for each participant from measurements at the nearest PM_2.5_ monitor within either 8 or 30 km of her geocoded residential address. Each participant was assigned a single exposure value over the follow-up period, defined as the average pollutant concentration from the beginning of the observation period (1 June 2002) to the woman’s date of death, loss to follow-up, or study termination (31 July 2007). Thus, exposure assignment was dependent on the duration of follow-up for each participant.

In our article (Ostro et al. 2010), we reported associations of mortality from all causes, cardiopulmonary disease, and ischemic heart disease (IHD) with PM_2.5_ mass and several of its components. However, the estimated hazard ratios (HRs) were generally higher than those reported from previous cohort studies (Dockery et al. 1993; Eftim et al. 2008; Krewski et al. 2009; Laden et al. 2006; Pope et al. 1995). Part of this difference was likely due to the nature of the exposure assignment. Most previous cohort studies have assigned the same exposure period to all study subjects, regardless of when deaths occurred. Thus, estimated exposures for some study participants in several studies occurred after their deaths. In addition, exposures have usually been assigned to participants based on their residential address at enrollment only, without taking into account exposure changes that may have occurred throughout the study period or when participants relocated. Finally, many previous studies measured exposure for only a subset of the years during which the cohort was followed. In an effort to reduce these aspects of exposure misclassification, we estimated exposures beginning prior to the cohort follow-up period, continuing to the end of the study or until the participant died or relocated out of state, incorporating updated exposure assignments when the subjects moved.

Importantly, measured concentrations of several pollutants in California declined substantially from 2002 through 2007; annual average PM_2.5_, organic carbon (OC), and nitrates decreased by around 30% each. These marked decreases in ambient PM_2.5_ concentrations resulted in lower average exposure estimates for cohort members who survived to the end of our study. Thus, the exposure assigned to a participant who died at time *t* would tend to be greater for events occurring early in the observation period, compared with the long-term average exposures of the participants who comprised the remainder of the risk set (i.e., those who were still part of the cohort study at time *t* and who subsequently experienced lower ambient pollution levels).

We have reanalyzed the CTS data using time-dependent pollution metrics—in which the exposure estimates for everyone remaining alive in the risk set were recalculated at the time of each death—in order to compare their average exposures up to that time with that of the individual who had died. In this way, decedents and survivors comprising the risk set had similar periods of pollution exposure, without subsequent pollution trends influencing the surviving women’s exposure estimates.

As in our previous study (Ostro et al. 2010), we restricted the sample in this reanalysis to women living within 30 km of one of eight fixed-site monitors in the U.S. Environmental Protection Agency’s Speciation Trend Network (STN), resulting in a study population of almost 44,000 women. Residential addresses from study enrollment forward were geocoded and linked with monthly pollutant averages at the nearest STN monitor to generate estimates of long-term exposure. We also used the same set of individual and ecological covariates in a Cox proportional hazards model as was used in the original study. Pollutants entered separately into the model included PM_2.5_ mass, elemental carbon (EC), OC, sulfate, nitrate, iron, potassium, silicon, and zinc. We used data on primary cause of death from August 2002 through July 2007 to examine the relationships between pollutants and mortality from all causes and cardiopulmonary, pulmonary, and IHDs.

The results are summarized in Erratum Table 1, scaled to the interquartile range (IQR) for each pollutant. HRs were significantly attenuated from our previous results. No associations were observed between all-cause mortality and PM_2.5_ or its components. For cardiopulmonary mortality, we observed significant associations for PM_2.5_ mass, nitrate, sulfate, and silicon, with more modest associations for zinc. PM_2.5_ mass and all of its components were associated with mortality from IHD, whereas none of the pollutants was associated with pulmonary mortality. This Erratum Table 1 should replace Table 5 in our previous article (Ostro et al. 2010).

Compared with our previous results (Ostro et al. 2010), these updated PM_2.5_ HRs are more consistent with several other published estimates of mortality risks, which are scaled to an increment of 10 μg/m^3^ of long-term average PM_2.5_ and summarized in Erratum Table 2. For example, relative to our revised HR of 1.19 for cardiopulmonary disease, analogous HRs from previous studies include 1.09 (95% CI, 1.03–1.16) from the American Cancer Society–Cancer Prevention II (ACS) cohort (cardiopulmonary disease; Pope et al. 2004), 1.28 (95% CI, 1.13–1.44) from the Harvard Six Cities study (cardiovascular disease; Laden et al. 2006), and 1.10 (95% CI, 0.94–1.28) from the Los Angeles subcohort of the ACS study (cardiopulmonary disease; Jerrett et al. 2005). Much higher HRs were observed in the observational study of the Women’s Health Initiative cohort for cardiovascular and IHD mortality (Miller et al. 2007).

These revised results still support the existence of elevated risks of PM_2.5_-associated cardiopulmonary disease and IHD, and illustrate the importance of considering the impact of long-term pollution trends in modeling estimates of exposure.

## Figures and Tables

**Erratum Table 1:** Association between mortality outcomes and PM_2.5_ and its components using a 30-km buffer (*n* = 43,220).

Pollutant	IQR (μg/m^3^)	All-cause (ICD-10 codes, all except S–Z) (*n* = 2,519)		Cardiopulmonary (ICD-10 codes, I00–I99, J00–J98) (*n* = 1,357)		Ischemic heart disease (ICD-10 codes, I20–I25) (*n* = 460)		Pulmonary (ICD-10, codes J00–J98) (*n* = 355)	
		HR (95% CI)	*p*-Value	HR (95% CI)	*p*-Value	HR (95% CI)	*p*-Value	HR (95% CI)	*p*-Value
PM_2.5_	6.1	1.03 (0.98–1.10)	0.26	1.11 (1.03–1.21)	0.01	1.31 (1.14–1.50)	< 0.01	1.02 (0.87–1.19)	0.84

EC	0.65	1.02 (0.93–1.12)	0.65	1.07 (0.94–1.22)	0.28	1.46 (1.17–1.83)	< 0.01	0.88 (0.68–1.15)	0.35

OC	0.84	1.00 (0.95–1.04)	0.91	1.04 (0.98–1.11)	0.19	1.13 (1.01–1.25)	0.03	0.95 (0.84–1.06)	0.35

Sulfate	2.2	1.06 (0.97–1.16)	0.18	1.14 (1.01–1.29)	0.03	1.48 (1.20–1.82)	< 0.01	1.04 (0.82–1.31)	0.77

Nitrate	3.2	1.03 (0.98–1.09)	0.27	1.11 (1.03–1.19)	0.01	1.27 (1.12–1.43)	< 0.01	1.04 (0.90–1.20)	0.58

Iron	0.13	1.01 (0.93–1.11)	0.77	1.05 (0.93–1.19)	0.40	1.39 (1.13–1.72)	< 0.01	0.88 (0.69–1.13)	0.32

Potassium	0.07	1.01 (0.94–1.08)	0.85	1.06 (0.97–1.17)	0.22	1.27 (1.07–1.49)	< 0.01	0.90 (0.74–1.09)	0.27

Silicon	0.03	1.02 (0.99–1.06)	0.22	1.05 (1.00–1.10)	0.04	1.11 (1.02–1.20)	0.01	0.98 (0.89–1.08)	0.71

Zinc	0.01	1.03 (0.96–1.11)	0.45	1.09 (0.98–1.20)	0.10	1.33 (1.12–1.58)	< 0.01	0.97 (0.79–1.18)	0.74

ICD-10, *International Classification of Diseases,* 10th Revision (World Health Organization 1993). Values shown are HRs [95% confidence intervals (CIs)] and *p*-values scaled to the IQR of each pollutant. All models are adjusted for smoking status, total pack-years, body mass index, marital status, alcohol consumption, second-hand smoke exposure at home, dietary fat, dietary fiber, dietary calories, physical activity, menopausal status, hormone replacement therapy use, family history of myocardial infarction or stroke, blood pressure medication and aspirin use, and neighborhood contextual variables (income, income inequality, education, population size, racial composition, unemployment).

**Erratum Table 2:** Comparative HRs (95% CIs) associated with a 10-μg/m^3^ change in long-term exposure to PM_2.5_ in several cohort studies conducted in the United States.

Authors	Exposure assessment	All causes	Cardiovascular	Cardiopulmonary	IHD
Ostro et al. (this report)	From 1 year prior to follow-up until event (either death or end of study), time-dependent	1.06 (0.96–1.16)^a^		1.19 (1.05–1.36)^a^	1.55 (1.24–1.93)^a^
Ostro et al. (2010)	From 2 months prior to study through event month	1.84 (1.66–2.05)^a^		2.05 (1.80–2.36)^a^	2.89 (2.27–3.67)^a^
Pope et al. (2002, 2004)	Four years prior to or at start of follow-up and 2 years after end of follow-up	1.06 (1.02–1.11)	1.12 (1.08–1.15)	1.09 (1.03–1.16)	
Laden et al. (2006)	Multiyear average concurrent with follow-up	1.16 (1.07–1.26)	1.28 (1.13–1.44)		
Miller et al. (2007)	One year in middle of follow-up		1.76 (1.25–2.47)^a^		2.21 (1.17–4.16)^a^
Jerrett et al. (2005)	One year at end of follow-up	1.15 (1.03–1.29)		1.10 (0.94–1.28)	1.32 (1.05–1.66)
Eftim et al. (2008)	Three-year average concurrent with follow-up	1.21 (1.15–1.27)			
Chen et al. (2005)	Four-year moving average prior to event				1.42 (1.06–1.90)^a^
Puett et al (2009)	One year prior to event	1.26 (1.02–1.54)^a^			2.02 (1.07–3.78)^a^

HRs are scaled to 10-μg/m^3^ change in PM_2.5_, in contrast to Table 1, in which HRs are scaled to the pollutant interquartile range.

a Women only.

## References

[b1-ehp-119-a242] Chen LH, Knutsen SF, Shavlik D, Beeson WL, Petersen F, Ghamsary M (2005). The association between fatal coronary heart disease and ambient particulate air pollution: are females at greater risk?. Environ Health Perspect.

[b2-ehp-119-a242] Dockery DW, Pope CA, Xu X, Spengler JD, Ware JH, Fay ME (1993). An association between air pollution and mortality in six U.S. cities. N Engl J Med.

[b3-ehp-119-a242] Eftim SE, Samet JM, Janes H, McDermott A, Dominici F (2008). Fine particulate matter and mortality: a comparison of the Six Cities and American Cancer Society cohorts with a Medicare cohort. Epidemiology.

[b4-ehp-119-a242] Jerrett M, Burnett RT, Ma R, Pope CA, Krewski D, Newbold KB (2005). Spatial analysis of air pollution and mortality in Los Angeles. Epidemiology.

[b5-ehp-119-a242] Krewski D, Jerrett M, Burnett RT, Ma R, Hughes E, Shi Y (2009). Extended Follow-Up and Spatial Analysis of the American Cancer Society Study Linking Particulate Air Pollution Aand Mortality. HEI Research Report 140.

[b6-ehp-119-a242] Laden F, Schwartz J, Speizer FE, Dockery DW (2006). Reduction in fine particulate air pollution and mortality: extended follow-up of the Harvard Six Cities study. Am J Respir Crit Care Med.

[b7-ehp-119-a242] Miller KA, Siscovick DS, Sheppard L, Shepherd K, Sullivan JH, Anderson GL (2007). Long-term exposure to air pollution and incidence of cardiovascular events in women. N Engl J Med.

[b8-ehp-119-a242] Ostro B, Lipsett M, Reynolds P, Goldberg D, Hertz A, Garcia C (2010). Long-term exposure to constituents of fine particulate air pollution and mortality: results from the California Teachers Study. Environ Health Perspect.

[b9-ehp-119-a242] Pope CA, Burnett RT, Thun MJ, Calle EE, Krewski D, Ito K (2002). Lung cancer, cardiopulmonary mortality, and long-term exposure to fine particulate air pollution. JAMA.

[b10-ehp-119-a242] Pope CA, Burnett RT, Thurston GD, Thun MJ, Calle EE, Krewski D (2004). Cardiovascular mortality and long-term exposure to particulate air pollution: epidemiological evidence of general pathophysiological pathways of disease. Circulation.

[b11-ehp-119-a242] Pope CA, Thun MJ, Namboodiri MM, Dockery DW, Evans JS, Speizer FE (1995). Particulate air pollution as a predictor of mortality in a prospective study of U.S. adults. Am J Respir Crit Care Med.

[b12-ehp-119-a242] Puett RC, Hart JE, Yanosky JD, Paciorek C, Schwartz J, Suh H (2009). Chronic fine and coarse particulate exposure, mortality and coronary heart disease in the Nurses’ Health Study. Environ Health Perspect.

[b13-ehp-119-a242] World Health Organization (1993). International Classification of Diseases, 10th Revision.

